# Omics Analysis of Lignin Degradation by the Gut Microbiomes of Wood‐Eating *Hypomeces squamosus* Fabricius

**DOI:** 10.1002/mbo3.70208

**Published:** 2025-12-28

**Authors:** Chunlan Mao, Qing Zhang, Jing Zhang, Xiangkai Li

**Affiliations:** ^1^ State Key Laboratory of Ecological Safety and Sustainable Development in Arid Lands, Northwest Institute of Eco‐Environment and Resources Chinese Academy of Sciences Lanzhou China; ^2^ Lanzhou Eco‐Agriculture Experimental Research Station Lanzhou China; ^3^ Ministry of Education Key Laboratory of Cell Activities and Stress Adaptations School of Life Science Lanzhou University Lanzhou China

**Keywords:** enzyme‐encoding genes, gut microbiomes, lignin degradation

## Abstract

Microbial degradation of lignin is important to carbon cycling. The gut microbiome of wood‐feeding *Hypomeces squamosus* Fabricius has been shown to degrade lignin efficiently. However, the specific degradation mechanisms remain incompletely understood. In this study, we investigated the mechanism of lignin degradation using omics comparative analysis, focusing on differentially expressed genes and metabolic pathways in the gut microbiome of insects fed with a lignin‐rich diet. The dominant genus taxon was *Pantoea* (29.82%), which was predominant in insects fed with high lignin‐containing *Iris ensata* Thunberg, whereas *Wolbachia* and *Enterobacter* were predominant in insects fed with cabbage leaves (MHS_K group). Furthermore, expression levels of carbohydrate‐active enzymes from the auxiliary activities (AAs) families in the MHS_I group were 1.18 times higher than those in the MHS_K group. These mainly included lignin peroxidase and manganese peroxidase of the AA2 family, vanillyl‐alcohol oxygenase of the AA4 family, and 1,4‐benzoquinone reductase of the AA6 family. Expression levels of multiple genes encoding aromatic compound‐degrading genes (2303 accounted for 75.76% of the total upregulated genes) were found, including about 0.03% was related to lignin degradation. Genes MHS‐HN_11398_2 (protocatechuate 2,3‐dioxygenase) and MHS‐HN_4821_1 (muconolactone d‐isomerase) were enriched in the MHS_I group. Three lignin‐degrading pathways were found: ortho‐cleavage and meta‐cleavage of catechol, as well as ring‐opening of protocatechuate. This study provides a comprehensive and theoretical evidence of the gut microbiome roles of *H. squamosus* Fabricius in lignin degradation.

## Graphical abstract

(1) The dominant lignin‐degrading genus taxon was *Pantoea*. (2) The key genes MHS‐HN_11398_2 (protocatechuate 2,3‐dioxygenase) and MHS‐HN_4821_1 (muconolactone d‐isomerase) were identified. (3) Three degrading pathways, ortho‐cleavage and meta‐cleavage of catechol, and ring‐opening of protocatechuate, were found.

## 1 Introduction

Lignocellulose, the largest carbon pool in the world, plays an important role in keeping the carbon balance of the Earth. With the development of the Global Carbon Project, the degradation and utilization of lignocellulosic biomass have attracted the extensive attention of scholars (Lubbers et al. 2019). Lignin is a phenylpropanoid polymer and a key component that endows vascular plants with secondary cell wall toughness and high mechanical strength. It complicates decomposition and utilization of lignocellulose under normal conditions (Tarmadi et al. 2018). Conventional lignin pretreatment processes usually require extreme temperatures, pH, and pressures, requiring high energy expenditure and large capital investments (Tsegaye et al. 2018). Microorganisms, including fungi and bacteria, can be utilized to degrade lignin efficiently, at a low cost and without pollution (R. Xu et al. 2018). White rot fungi, such as *Phanerochaete chrysosporium*, are known for their ability to degrade lignin (Chen and Wan 2017). However, challenges in fungal protein expression and genetic manipulation limit the commercial use of this fungus. Therefore, lignin‐degrading bacteria have received increasing attention owing to their environmental adaptability and potential for industrial applications (Bugg et al. 2011). Currently, lignin‐decomposing strains are mainly screened from soil, rotten wood, and the guts of wood‐feeding insects. *Pseudomonas*, *Bacillus*, and *Acinetobacter* are examples of bacteria with high lignin degradation efficiency.

Wood‐eating insects have evolved to digest a wide range of lignocellulosic substrates. Therefore, their guts are natural reservoirs of lignin‐degrading microorganisms. Currently, the gut of wood‐feeding termites is the most widely studied natural biomass degradation system (Geng et al. 2018; H. Zhou et al. 2017; Sousa et al. 2019). Given that wood is their sole food source, termites efficiently digest wood lignocellulose within a relatively short period (Ke et al. 2011). The termite gut microbiome symbionts digest cell wall polysaccharides and protozoa in the hindgut using various cellulose‐degrading enzymes (Luo et al. 2019a). Furthermore, the efficiency of the degradation of conjugated aromatic structures in the foregut and midgut of termites was higher than that in the hindgut (Ke et al. 2011). In other studies, insects such as longhorned beetle, bamboo snout beetle, and locusts, which fed on plants with high lignin content, were also identified as decomposers (Pauchet et al. 2014; Akcay and Yalcin 2021). The guts of all these insects express genes encoding enzymes, such as xylanase, cellulase, and glycosyl hydrolase genes (Scully et al. 2012; Luo et al. 2018). These enzymes enable insects to digest a range of lignocellulosic feedstocks (Oppert et al. 2010). However, the specific degradation mechanisms and synergistic effects of gut microbes require further study. In general, the degradation of lignocellulose by a natural biomass degradation system is pollution‐free, environmentally friendly, and cost‐effective. Harnessing the natural mechanism of lignocellulose bioconversion can provide a basis for its industrial implementation (Xie et al. 2014).

*Hypomeces squamosus* Fabricius (Coleoptera: Curculionidae) is a highly destructive crop pest widely distributed in southern China. Its growth and reproduction occur from April to June. The adults show obvious clustering and prefer feeding on the new shoots and leaves of cotton seedlings, tea trees, and other plants. In serious cases, they also gnaw on the bark, affecting the vitality of the tree, sometimes killing the entire plant. To date, scarce reports on this insect have focused mainly on crop pest control and pest management (F. F. Xu et al. 2014; M. Y. Li et al. 2020). In our previous study, a lignin‐degrading strain was isolated from *H. squamosus* gut and used in anaerobic digestion (Q. Zhang et al. 2021). We showed the presence of lignin‐degrading strains in *H. squamosus* guts. Were there any other lignocellulose‐degrading microbial resources (including un/noncultured) in the *H. squamosus* gut? Did any genes encoding lignocellulose‐degrading enzymes exist? It was not yet clear. Furthermore, as a natural bioreactor of lignocellulose, over 90% gut microbiome is noncultured. Utilization of unexplored lignocellulolytic microbial resources is imperative due to their important role in carbon recycling. Different omics, such as metagenomics, metatranscriptomics, and metaproteomics (H. Zhou et al. 2017; Luo et al. 2019a, 2018; Scully et al. 2014), provided an effective method for evaluating the potential bacteria, function, and genes of insect gut flora. Therefore, bio‐prospection using omics‐based techniques could be of immense advantage in mitigating the quest for various novel key hydrolytic enzymes (Show et al. 2022) (unexplored microbial resources).

Therefore, the aim of this study was to investigate the lignocellulose‐degrading microbial community, metabolic pathways, and functional genes/enzymes in the gut of *H. squamosus* using omics analysis. Total RNA was extracted from the insect gut fed with different diets and subjected to high‐throughput RNA sequencing. Transcriptome analysis showed that the expression levels of genes encoding the auxiliary activity (AA) family of carbohydrate‐active enzymes (CAZymes) in insects fed with the high lignin group were significantly higher than those in the low lignin diet group. The present study focused on the changes in gut microflora and expression of genes encoding ligninolytic enzymes. The differentially expressed components of metabolic pathways and genes were further analyzed to reveal the lignin degradation pathways of *H. squamosus* gut microorganisms. This study provided a comprehensive and theoretical evidence of the gut microbiome roles of *H. squamosus* Fabricius in lignin degradation.

## 2 Materials and Methods

### 2.1 Insect Collection and Treatment

Adult *H. squamosus* was collected from the Xiama Forest Farm, Tianzhu County, Gansu Province, China. The insects were divided into two groups that were fed with food containing different lignin contents for a week. The control group (CK) (MHS_K) was fed with cabbage containing 5% lignin, whereas the treatment group (TG) (MHS_I) was fed on *Iris lactea* Pall. belonged to *Iris* of Iridaceae with 24% lignin content. And the end of the experimental period, the guts were extracted, placed in RNA‐free centrifuge tubes, and immediately frozen in liquid nitrogen for subsequent sample delivery and sequencing.

### 2.2 Antibiotic Assay

To further verify that the lignin degradation was mainly achieved by the gut microbiome of H. squamosus Fabricius, the inhibition tests of antibiotics were set. A group of 60 *H. squamosus* insects fed with *I. lactea* Pall. as the sole diet for 1 week was collected. The insect surfaces were sterilized by soaking in 75% ethanol for 1 min, and then rinsed twice with sterile salt water. Next, their guts were extracted and collected in a 10‐mL centrifuge tube containing 5 mL of salt water. After shaking on a vortex mixer for 5 min, gut tissues were carefully removed using a pipette. The gut suspension was then used for a subsequent antibiotic screening test. Gentamicin was tested for its ability to inhibit *H. squamosus* gut bacteria using the inhibition halo test. The gut suspension (100 μL) was inoculated and spread across a plate with Luria–Bertani (LB) medium. Subsequently, discs containing 30 μg of gentamicin were placed on the surface of the inoculated plates. Antibiotic‐free discs served as controls. The culture plates were incubated in triplicate for 1 day at 37°C, and then the inhibition halos were measured.

In the next experiment, three groups of insects (160 in each group) were fed with the selected antibiotic diet (*I. lactea* Pall. sprayed with water containing 30 mg/L antibiotic) for 7 days, whereas the other three CKs were fed with normal *I. lactea* Pall, without antibiotics. On days 1, 3, 5, and 7, 30 insects were randomly collected from each group to prepare a gut suspension, as described above. Another 40 insects in each group were used to calculate daily survival rates. The suppression of gut bacteria was assessed based on the bacterial counts estimated using the serial dilution method of plate counting.

Another 120 *H. squamosus* insects were divided into three groups. After treatment with gentamicin for 3 days, the daily food consumption rate of each group was recorded. The CK in this experiment was treated without antibiotics.

### 2.3 DNA Extraction, Library Construction, and Sequencing

The OMEGA stool DNA Kit (OMEGA, Shanghai, China) was used to extract total metagenomic DNA from the gut microflora. Subsequently, an inserted fragment library of the appropriate length was constructed after random interruptions into short fragments. These libraries were based on the high‐throughput Illumina HiSeq sequencing platform, and paired‐end sequencing was performed using the Whole‐Genome Shotgun method.

### 2.4 RNA Extraction, Complementary DNA (cDNA) Library Construction, and RNA Sequencing

Total RNA was extracted from the gut samples using the OMEGA stool RNA Kit (OMEGA, Shanghai, China). After Ribo‐zero ribosomal RNA (rRNA) Removal Kit (Epicenter, WI, USA) was used to remove rRNA from the environmental samples, 1% agarose gel electrophoresis was used to detect the extracted RNA. Ion‐breaking reagents and random primers were added to the extracted RNA, which was fragmented, so that the random primers could bind to the fragments. The cDNA library was constructed after reverse transcription into cDNA, and sequencing was performed using Illumina NovaSeq by Shanghai Biozeron Co.

### 2.5 Gene Annotation and Functional Analysis of Differentially Expressed Genes

META Prodigal was used to predict gene sequences in all samples. The protein sequences of the predicted genes were compared with the nonredundant protein collection (NCBI‐NR), Evolutionary Genealogy of Genes: Nonsupervised Orthologous Groups (EggNOG) v5.0 (http://eggnog.embl.de), CAZy Database, and Kyoto Encyclopedia of Genes and Genomes (KEGG) (https://www.kegg.jp/) Orthology to obtain the annotation information. R package Edger was used to analyze the significance of differences in gene abundance between groups. Genes with significantly different expression levels were identified according to the following criteria: false discovery rate ≤ 0.05 and fold change ≥ 2. The KEGG database was used to classify the differentially expressed genes according to the pathways or functions they were involved in. The differentially expressed genes were visualized using the KEGG pathway map.

### 2.6 CAZyme Family Analysis in the Transcriptome

To identify related enzymes involved in lignocellulose degradation, nonredundant protein sequences were searched and analyzed in dbCAN (database) for automated CAZyme annotation using HMMER3 based on Profile Hidden Markov Models (Yin et al. 2012). The results of the functional annotation of each protein were summarized and statistically analyzed to obtain information about the abundance of the following members of the CAZy family: glycoside hydrolases (GHS), glycosyl transferases, polysaccharide lyases (PLS), carbohydrate esterases (CES), AAs, and carbohydrate‐binding modules.

### 2.7 Statistical Analysis

The number of gut bacteria (colony‐forming units, CFUs), insect survival rate, and insect food consumption rate after antibiotic treatment were expressed as the mean ± standard deviation. Statistical software (SPSS 20.0) was used for the data analysis. One‐way analysis of variance was used to compare the survival rates of *H. squamosus* treated with antibiotics for different periods and daily food consumption rates after 3 days of antibiotic treatment. All graphs were plotted using GraphPad Prism 6.

## 3 Results and Discussion

### 3.1 Effect of Antibiotics on Gut Bacteria

Gentamicin is a commonly used antibiotic that exerts its bactericidal action by irreversibly binding the 30S subunit of bacterial ribosomes to interrupt protein synthesis. Therefore, gentamicin was selected for the inhibition of gut bacteria (Yang et al. 2020) to verify their role in lignin degradation.

**Figure 1:**
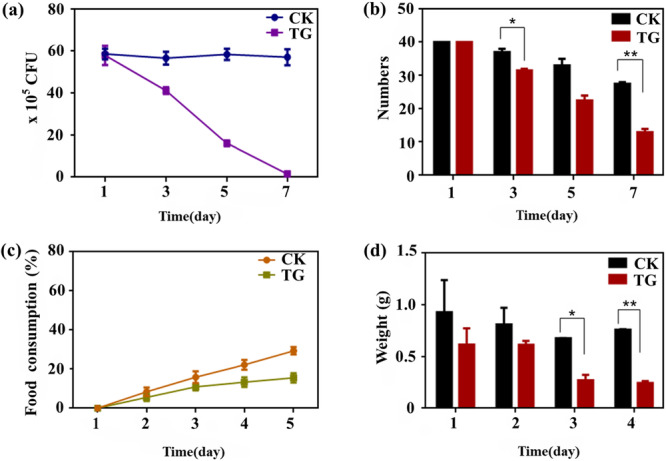
Contribution of gut bacteria to lignin degradation. (a) Total number of gut bacteria in insects treated with gentamicin for 1 week. (b) The number of insect survivors treated with gentamicin on different days. (c) Food consumption rate and (d) daily intake of insects after 3 days of antibiotic treatment. Statistical analysis was performed by Student's *t* test. *p < 0.05. CFU, colony‐forming unit; CK, control group; TG, treatment group.

Gentamicin potently suppressed the growth of gut bacteria on LB medium and formed clear inhibition halos (Figure S1). Estimation of the number of gut bacteria by serial dilution and plate counting showed that gut bacteria were significantly inhibited (Figure 1a). After 1 week, the number of CFUs in the LB medium inoculated with the gut suspension was close to zero, indicating that gut bacteria were essentially eliminated by the treatment with gentamicin. Figure 1b showed the number of *H. squamosus* insects that survived for the indicated number of days in the antibiotic TG and CK. Compared with the survival rates in the CK, the survival rate in the TG decreased gradually with time, with a significant difference between survival rates at days 3 and 7. These results showed that gut microbes were sensitive to gentamicin. The food consumption rate and daily intake of the insects were calculated after 3 days, as shown in Figure 1c,d. The daily intake and food consumption rate of the insects in the TG gradually decreased, whereas no significant change was observed in the CK group. This indicates that the microbes were cleared by the antibiotic, which affected the digestion and degradation of lignin. These results illustrate that the lignin‐degrading ability of *H. squamosus* mainly derives from its gut microbes. Similarly, the gut microbiota of the Asian Longhorned beetle (*Anoplophora glabripennis*) could degrade lignin in the woody diet to provide essential nutrients for the beetle (Scully et al. 2014). The gut microbe of wood‐feeding *Odontotaenius disjunctus* depolymerized lignocellulose in the mid‐hindgut of the host to provide energy and nutrition for its survival and development (Ceja‐Navarro et al. 2019). These results are consistent with the observation in this study that gut microbes of wood‐eating *H. squamosus* participated in the digestion and degradation of lignocellulose in the diet.

### 3.2 Characteristics and Functional Analysis of Gut Microbiomes of *H. squamosus*

#### 3.2.1 Metagenomic Sequencing and Assembly

Using the Whole‐Genome Shotgun strategy based on the Illumina HiSeq high‐throughput sequencing platform, total DNA extracted from bacterial metagenomes was randomly fragmented into short sequences, and inserts of appropriate length were constructed. A total of 15 libraries were constructed and subjected to paired‐end sequencing. Effective sequences were filtered after quality control of the sequencing data. Then, the assembly and splicing of metagenomic sequences and the construction of nonredundant sequences were performed. The MHS_K group and MHS_I group had 962,747 and 1,409,136, spliced sequences, respectively. The detailed metagenomic assembly and splicing data were presented in Table S1.

#### 3.2.2 Microbial Community Structure

To investigate the diversity of *H. squamosus* gut microbes, the scaffold sequences of each sample were compared with the sequences of Bacteria, Archaea, Fungi, and Viruses in the NCBI‐NT database using BLASTN (*E* < 0.001). In addition, the “Lowest Common Ancestor (LCA)” algorithm (Huson et al. 2007), implemented in MEGAN software (Willis et al. 2017), was used to assign taxonomic classifications to the target sequences. Specifically, the most specific taxonomic node shared by all reference sequences matching a query was identified, which served as the species‐level annotation for the target sequence.

**Figure 2:**
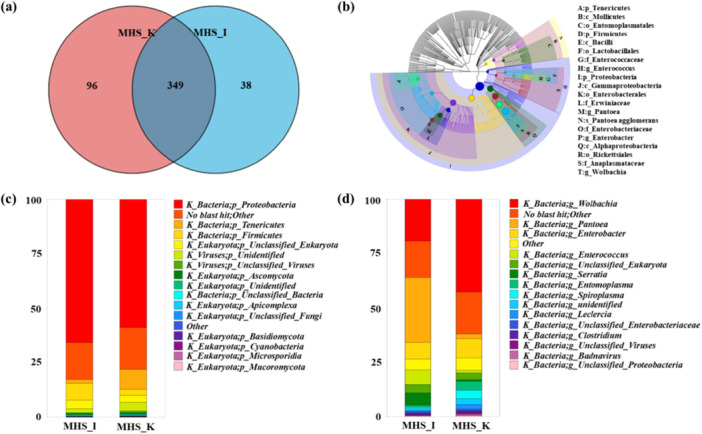
Structure of microbial community in *Hypomeces squamosus* Fabricius. (a) Venn diagram of two groups of common species. (b) Overall diagram of the sample population classification level tree. Metagenomic community composition and structure at (c) phylum level and (d) genus level. Statistical analysis was performed by Student's *t* test. *p < 0.05.

The detailed community diversity and structure were shown in Figure 2. A Venn diagram of the common taxa in the two groups was shown in Figure 2a. There were 349 common species, and the number of unique species in the MHS_I group was lower than that in the MHS_K group. This could have occurred owing to changes in the digestive bacterial flora after *H. squamosus* was fed cabbage leaves, which led to an increase in community diversity. However, according to the α‐diversity index (Table S2), the flora richness of the MHS_I group was higher than that of the MHS_K group. This indicates that the abundance of lignocellulose‐degrading bacteria in the MHS_I group was higher. A population classification tree was created using GraPhlAn to illustrate the taxonomic composition of the sample population at each taxonomic level. Different colors were used to distinguish each taxon, and their abundance distribution was reflected by the node size (Figure 2b). At the phylum level (Figure 2c), *Proteobacteria* was the dominant taxon in both groups. However, some of the bacteria could not be identified, which might be because no studies have been conducted on the gut flora of this insect. Therefore, further studies of the *H. squamosus* microflora are needed. At the genus level (Figure 2d), *Wolbachia* and *Enterobacter* had higher relative abundances in both the MHS_I group and MHS_K group. These are common symbiotic bacteria belonging to the order Coleoptera. *Pantoea* was the dominant taxon in the MHS_I group (29.82%). In previous studies, *Pantoea* strains were found in the gut of bark beetles and could be used to degrade plant cell components. They showed well activities for the degradation of lignocellulose, such as cellulose and xylan (Fabryová et al. 2018). Also, *Pantoea* showed extracellular hydrolase activity of cellulose, amylase, and protease (Suman et al. 2020). Therefore, as one of the gut microbiomes, *Pantoea* had lignocellulose‐degrading potential. In this study, the abundance of *Pantoea* in the MHS_I group was much higher than that in the MHS_K group, because insects fed with *Iris ensata* Thunberg. Could digest and degrade lignin. This also supports the idea that gut bacteria provide nutrients for *H. squamosus* by hydrolyzing lignocellulose.

#### 3.2.3 Functional Analysis of Microorganisms

**Figure 3:**
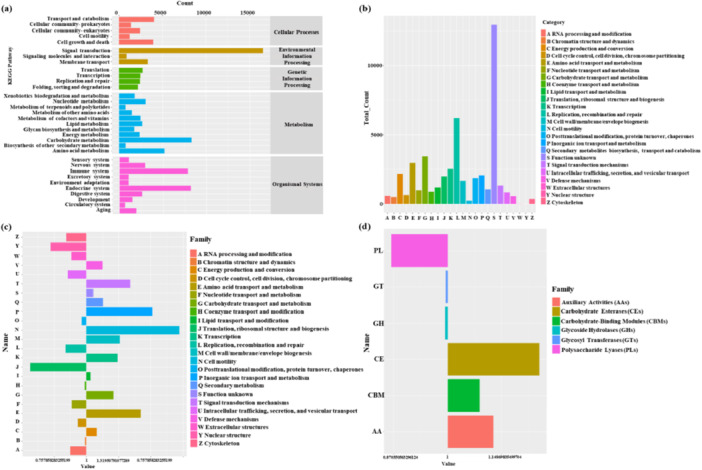
Functional annotations of metagenomic genes. (a) Statistical chart of annotation results of the KEGG pathway at the second level. (b) Annotated statistical graph of the EggNOG functional group. Comparison diagram of (c) NOG functional group and (d) CAZy protein functional module expression in the samples. KEGG, Kyoto Encyclopedia of Genes and Genomes.

To understand the functional roles of the *H. squamosus* gut microbiota, we focused on KEGG metabolic pathways and EggNOG functional annotation (Figure S2). The KEGG pathways were primarily concentrated in Metabolism, Genetic Information Processing, and Environmental Information Processing, Cellular Processes, and Organismal Systems. Horizontal coordinates represent the number of proteins annotated to the corresponding metabolic pathway. As shown in Figure S2a, the metabolic pathways were dominated by carbon metabolism (8329), amino acid metabolism (5191), lipid metabolism (2676), energy metabolism (2374), xenobiotic biodegradation and metabolism (1800), and glycan biosynthesis and metabolism (1754). Figure S2b presented a statistical graph of the annotation results of EggNOG functional taxa, with 26,908 proteins grouped into 26 functional categories. Among them, 27.62% was of unknown function, and 7.3% was related to carbohydrate transport and metabolism. Figure S2c and 3a illustrate the distribution of relative abundances of functional taxa at each level, based on the annotation of each sample in the EggNOG and CAZy functional databases. As shown in Figure S2c, the expression level of the G group (Transport and metabolism) in the MHS_I group was 1.26 times higher than that in the CK. Among genes encoding CAZymes, the expression level of the AA family was 1.18 times higher. Several enzymes secreted by microorganisms play an important role in lignin biodegradation (Luo et al. 2019b). According to previous reports, lignin is usually degraded by lignin peroxidase (AA2), manganese peroxidase (AA2), glucose methanol choline oxidoreductase (AA3), and laccase (AA1‐1) belonging to the AA family (Liu et al. 2019). The results showed that higher expression of AA family enzymes in the MHS_I group, which confirmed the expression of lignin‐degrading enzymes in the insect gut microbiome. It was also confirmed that the gut flora of this insect quickly evolved and adapted to the change in the diet to provide nutrition and energy for the host. At the same time, the host provides a suitable habitat and environment for the microorganisms. The host–microbe interactions complemented each other to support their mutual survival and evolution. A similar view was found in the gut of termites, where symbiotic microorganisms showed high efficiency lignocellulose degradation to polysaccharides for assimilation and energy (Tarmadi et al. 2018). Meanwhile, they could be used in biotechnological applications (cellulase enzymes) (J. Zhou et al. 2019). Additionally, in the insect gut, some resident microorganisms could promote insect fitness and protect them against pathogens and parasitoids (Douglas 2015). The number of bacteria expressing genes encoding the AA family enzymes was higher in the MHS_I group than in the MHS_K group (Figure 3b). The intergroup difference was particularly large for the genes of the AA6 family in *Pantoea alhagi*. After comparing the microflora = community structure in 2.2.1, it was found that, except for *P. alhagi*, *Lactococcus lactis*, and *Acinetobacter baumannii*, the other three bacteria could not be found. Therefore, it is speculated that these three bacteria may be nonculturable bacteria, and further research on them is still needed.

#### 3.2.4 Analysis of CAZyme Characteristics

In this study, 8185 CAZyme family proteins were annotated: GHS (3001), glycosyltransferases (3537), carbohydrate‐binding modules (675), CES (399), PLS (289), and AA enzymes (284). The relative abundances of the AA families associated with lignin degradation in the two groups and the corresponding protein numbers of each AA family were presented in Table 1. Expression levels of all AA family members in the MHS_I group were higher than in the MHS_K group. Expression levels of cellobiose dehydrogenase and aryl alcohol oxidase in the AA3 family were higher in both groups. It was similar to the transcriptome results of bamboo snout beetle *Cyrtotrachelus buqueti*, in which the expression levels of CAZymes in the insect gut upregulated after feeding on bamboo shoots (Luo et al. 2019a, 2018). Expression levels of lignin peroxidase and manganese peroxidase from the AA2 family, 1,4‐benzoquinone reductase from the AA6 family, and vanillyl‐alcohol oxidase from the AA4 family were significantly different. Lignin peroxidase and manganese peroxidase have been proven to be important and efficient lignin‐degrading enzymes, because they catalyze the initial steps of lignin depolymerization (Liu et al. 2019). Therefore, in the present study, lignin peroxidase, manganese peroxidase, 1,4‐benzoquinone reductase, and vanillyl‐alcohol oxidase were identified as the main lignin‐degrading enzymes. Various intermediate metabolites were produced, including benzoquinone, benzoic acid, other aromatic acids, vanillin, and products of ring‐opening reactions (Brock and Gold 1996). Quinones were the key intermediates in aromatic‐ring degradation. Benzoquinone reductase catalyzed the reduction of quinones to catechol. Therefore, benzoquinone reductase and vanillyl‐alcohol oxidase play important roles in lignin degradation (Lee et al. 2007). These results indicated that lignin‐degrading enzymes were expressed and secreted by the gut microorganisms of *H. squamosus*. Similarly, in the termite gut, a series of lignolytic and cellulolytic enzymes were secreted, such as laccase and lignin peroxidase for woody biomass degradation (Geng et al. 2018; H. Zhou et al. 2017). They showed strong activities toward hydroquinone and 2,6‐dimethoxypheno (Geng et al. 2018), dyes, lignin model monomers and dimers, and lignin sulfonate (H. Zhou et al. 2017; Ke et al. 2011). Therefore, lignin‐degrading bacteria presented in *H. squamosus* in the MHS_I group digested and degraded lignin into small molecular compounds to provide nutrition for the host. Especially, the midgut microbiome is enriched in biosynthetic pathways and synthesized essential amino acids, vitamins, and sterols to provide essential nutrients for the host insect or synthesizes itself (Scully et al. 2014).

**Table 1:** Predicted abundance of AA family and lignin‐degrading enzymes in the two groups.

Enzymes	CAZy modules	MHS_I (value)	MHS_K (value)	Protein (number)
Laccase (EC 1.10.3.2)	AA1	108.49	104.31	29
Lignin peroxidase (EC 1.11.1.14) Manganese peroxidase (EC 1.11.1.13)	AA2	44.67	14.51	7
Cellobiose dehydrogenase (EC 1.1.99.18) Aryl alcohol oxidase (EC 1.1.3.7)	AA3	734.02	733.63	222
Vanillyl‐alcohol oxidase (EC 1.1.3.38)	AA4	26.41	9.11	4
1,4‐Benzoquinone reductase (EC 1.6.5.6)	AA6	44.34	7.91	7
Glucooligosaccharide oxidase (EC 1.1.3.‐) Chitooligosaccharide oxidase (EC 1.1.3.‐)	AA7	27.02	14.49	6
Copper‐dependent lytic polysaccharide monooxygenases	AA10	39.26	20.02	8

Abbreviation: AA, auxiliary activity.

### 3.3 Screening of Differentially Expressed Genes for Lignin Degradation

#### 3.3.1 Total Sequences of Metatranscriptome and Metagenome

The original sequencing data were filtered to obtain high‐quality sequences, and ribosome sequences were removed to obtain clean data. MEGAHIT software was used for sequence splicing and assembly. After genetic prediction, 6613 and 37,666 complete open reading frames were obtained in the MHS_K group and MHS_I group, respectively, and their total sequence lengths were 3,853,863 and 18,549,478 bp, respectively. In the metagenome assembly, the total number of sequences (9,62,747) and the total number of bases (1,409,136) of the MHS_K group and MHS_I group were obtained, respectively. The lengths (total number of bases) of their metagenomes were 8.83E + 08 and 9.55E + 08 bp, respectively (Table S1). The lengths of genes in the metagenomic library were longer than those in the metatranscriptome library. Thus, the full‐length sequences of the differentially expressed genes in the metatranscriptome library could be obtained from the metagenomic library.

#### 3.3.2 Analysis of the Differentially Expressed Genes

**Figure 4:**
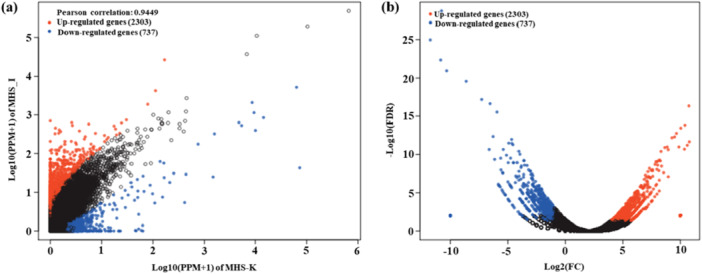
Analysis of differentially expressed genes. (a) Visual scatter plot and (b) volcano plot of differential genes (FDR ≤ 0.05 and FC ≥ 2). FC, fold change; FDR, false discovery rate.

EdgeR software was used to analyze significant differences in the abundance of each gene between the MHS_K and MHS_I groups. The scatter diagram of the differentially expressed genes (Figure 4a) and volcano plots (Figure 4b) showed that the MHS_K and MHS_I groups differed in the expression levels of 3040 genes, of which 2303 genes (75.76%) were upregulated and 737 genes (24.44%) were downregulated (Figure 4c). Furthermore, major lignin‐degrading enzymes accounted for ~ 0.03% of the upregulated genes. The specific distribution ratio was shown in Figure 4d. Genes encoding acetaldehyde dehydrogenase (EC 1.2.1.10 and EC 1.1.1.1) accounted for the highest proportion of 63.68%. Acetaldehyde dehydrogenase plays an important role in the degradation of aromatic compounds (Omokoko et al. 2008). Among the significantly upregulated genes, those related to lignin degradation and their corresponding functions were listed in Table 2. It found that protocatechuate 3,4‐dioxygenase, beta subunit (EC 1.13.11.3), and acetaldehyde dehydrogenase (EC 1.2.1.10 and EC 1.1.1.1) were enriched, which contributed to lignin degradation. The same results were also found in the lignin degradation by *Klebsiella pneumoniae* PX14 isolated from *C. buqueti* gut (Y.‐Q. Li et al. 2023). Therefore, the present study could well predict and evaluate the lignolytic enzymes in *H. squamosus* gut using genome data. Various conserved metabolic intermediates derived from lignin polymers, including catechol and protocatechuate, are important resources for the production of renewable aromatic compounds (Johnson and Beckham 2015). The degradation and utilization of these intermediates are the key steps in the depolymerization of lignin polymer (Mycroft et al. 2015). The ring‐opening of protocatechuic acid depends on the action of protocatechuate 2,3‐dioxygenase or protocatechuate 4,5‐dioxygenase (Kasai et al. 2007). Protocatechuate 2,3‐dioxygenase (gene MHS‐HN_11398_2, Table 2) participated in the degradation of both polycyclic aromatic hydrocarbons and aromatic compounds. Muconolactone d‐isomerase (gene MHS‐HN_4821_1) played an important role in the ring‐opening degradation of catechol. In addition, dehydrogenases encoded by other differentially expressed genes were involved in related pathways. These results suggested that the differentially expressed genes play an important role in lignin degradation.

**Table 2:** Significantly upregulated lignin degradation genes and related pathways.

Gene	Category	Pathway	Description
MHS‐HN__11398_2	Polycyclic aromatic hydrocarbon degradation	ko00624	pcaH; protocatechuate 3,4‐dioxygenase, beta subunit (EC 1.13.11.3)
MHS‐HN__11398_2	Degradation of aromatic compounds	ko01220	pcaH; protocatechuate 3,4‐dioxygenase, beta subunit (EC 1.13.11.3)
MHS‐HN__267_1	Degradation of aromatic compounds	ko01220	adhP; alcohol dehydrogenase, propanol‐preferring (EC 1.1.1.1)
MHS‐HN__980_1	Degradation of aromatic compounds	ko01220	E3.1.1.17, gnl, RGN; gluconolactonase (EC 3.1.1.17)
MHS‐HN__1703_1	Degradation of aromatic compounds	ko01220	adhE; acetaldehyde dehydrogenase/alcohol dehydrogenase (EC 1.2.1.10 and EC 1.1.1.1)
MHS‐HN__1860_1	Degradation of aromatic compounds	ko01220	adhE; acetaldehyde dehydrogenase/alcohol dehydrogenase (EC 1.2.1.10 and EC 1.1.1.1)
MHS‐HN__2519_1/MHS‐XM__29921_1	Degradation of aromatic compounds	ko01220	frmA, ADH5, adhC; *S*‐(hydroxymethyl)glutathione dehydrogenase/alcohol dehydrogenase (EC 1.1.1.284 and EC 1.1.1.1)
MHS‐HN__3624_1	Degradation of aromatic compounds	ko01220	adhE; acetaldehyde dehydrogenase/alcohol dehydrogenase (EC 1.2.1.10 and EC 1.1.1.1)
MHS‐HN__4821_1	Degradation of aromatic compounds	ko01220	catC; muconolactone d‐isomerase (EC 5.3.3.4)

Abbreviation: RGN, RNA‐guided nuclease.

#### 3.3.3 Differentially Expressed Metabolic Pathways

**Figure 5:**
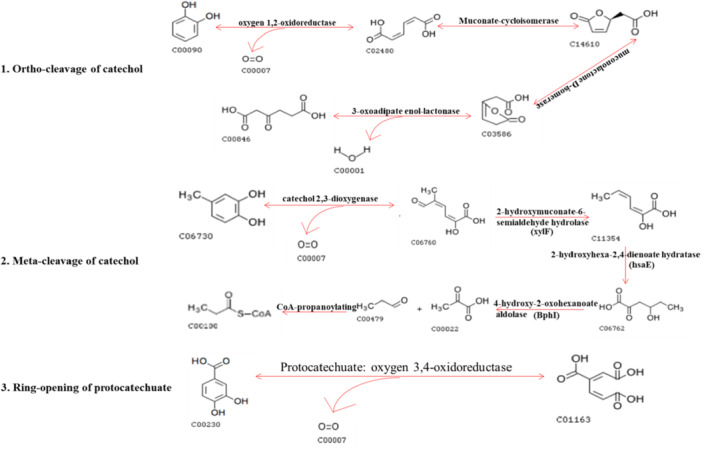
The three metabolic pathways related to lignin degradation excavated from the KEGG annotation pathway map of differential genes (FDR ≤ 0.05 and FC ≥ 2). KEGG, Kyoto Encyclopedia of Genes and Genomes. FC, fold change; FDR, false discovery rate

Using the KEGG database, genes were classified according to the pathways or functions performed by the proteins they encode. The differentially expressed genes in the two groups were displayed on the KEGG Pathway map. Figure 5 illustrates three metabolic pathways related to lignin degradation extracted from the KEGG annotation pathway map of the differentially expressed genes. These included ortho‐cleavage and meta‐cleavage of catechol and ring‐opening of protocatechuate. During ortho‐cleavage, catechol was oxidized to *cis*‐muconate by oxygen 1,2‐oxidoreductase, then 2‐oxo‐2,3‐dihydrofuran‐5‐acetate was generated under the action of muconate cyclooxygenase and muconolactone d‐isomerase, and it was finally oxidized to 3‐oxoadipate by 3‐oxodipate enol‐lactonase. During meta‐cleavage of catechol, the meta position of methylcatechol was broken by catechol 2,3‐dioxygenase. After ring‐opening, a series of enzymatic reactions produced small molecules (acetyl‐CoA, propionyl‐CoA, and pyruvate). In the protocatechuate pathway, protocatechuate 3,4‐dioxydoreductase opened the ring generating *cis*‐muconate, which then entered benzoate degradation pathway.

According to previous studies, lignin degradation can be divided into two stages (Dos Santos Melo‐Nascimento et al. 2020). The first stage is the preliminary depolymerization of lignin high polymers by lignin‐modifying enzymes, such as peroxidase, laccase, and manganese peroxidase, which correspond to the relevant enzymes of the AA family described in Section 3.2.3. In the second step, lignin‐derived aromatic intermediates are further decomposed by oxidases, monooxygenases, divalent oxygenases, dehydrogenases, and reductases. This corresponds to the series of enzymes described in Section 3.3.2. Lignin degradation pathways, including β‐aryl ether degradation pathway, ferulic acid metabolism, and oxidative decomposition of protocatechuate, have been extensively described (Abdelaziz et al. 2016; Davis and Sello 2010). The catechin and protocatechuate metabolic pathways described above have been verified by numerous previous studies. Our results showed that lignin‐degrading bacteria in the gut of *H. squamosus* secreted many enzymes that gradually depolymerized lignin into small molecular carbohydrates. These products of lignin biotransformation could likely be utilized during the growth and reproduction of *H. squamosus*.

## 4 Conclusions

*H. squamosus* Fabricius is a recognized crop pest in southern China. Current research has primarily focused on the prevention and control of this insect. However, as we have demonstrated previously, the gut of *H. squamosus* is a natural resource of microbes able to degrade lignin. The objects of this study were to investigate the lignin‐degrading microorganisms, determine expression levels of genes encoding relevant enzymes, and identify metabolic pathways important for lignin degradation in the gut microbiome of *H. squamosus*. Metagenomics was used to analyze the composition and structure of gut microbial communities and annotate the genes encoding lignocellulose‐degrading enzymes (CAZymes). Results showed that upon host consumption of a diet with high lignin content, microorganisms able to degrade lignin in the gut of *H. squamosus* were enriched, and expression levels of genes encoding enzymes contributing to lignocellulosic degradation. The results of the metatranscriptome analysis revealed significantly differentially expressed genes and metabolic pathways. Three metabolic pathways related to the lignin‐derived intermediates were identified. Although no strains or genes related to lignin degradation were isolated, the present study showed the great potential and rich microbial resources of the *H. squamosus* gut microbiome for lignin degradation.

## Author Contributions

**Chunlan Mao:** writing – original draft preparation, data curation, funding acquisition, writing – review and editing. **Qing Zhang:** supervision, data curation. **Jing Zhang:** software, formal analysis. **Xiangkai Li:** methodology.

## Ethics Statement

The authors have nothing to report.

## Conflicts of Interest

None declared.

## Supporting information


**Table S1:** Statistical table of metagenomics and meta‐transcriptome assembly data. **Table S2:** α‐diversity analysis index of Metagenomic. **Figure S1:** Inhibitory effect of gentamicin on the growth of bacteria extracted from the intestine of *Hypomeces squamosus* Fabricius. **Figure S2:** Functional annotations of metagenomic genes. (a) Statistical chart of annotation results of KEGG pathway at second level. (b) Annotated statistical graph of the EggNOG functional group. Comparison diagram of (c) NOG functional group in the samples.

## Data Availability

The data that support the findings of this study are available from the corresponding author upon reasonable request.
