# Supplementary material for: Omics Analysis of Lignin Degradation by the Gut Microbiomes of Wood‐Eating Hypomeces squamosus Fabricius
Source: Microbiologyopen. 2025 Dec 28;14(6):e70208. doi: 10.1002/mbo3.70208 (PMC12744954; doi:10.1002/mbo3.70208)
Supplement: Supplementary file 1 — Table S1: Statistical table of metagenomics and meta‐transcriptome assembly data. Table S2: α‐diversity analysis index of Metagenomic. Figure S1: Inhibitory effect of gentamicin on the growth of bacteria extracted from the intestine of Hypomeces squamosus Fabricius. Figure S2: Functional annotations of metagenomic genes. (a) Statistical chart of annotation results of KEGG pathway at second level. (b) Annotated statistical graph of the EggNOG functional group. Comparison diagram of (c) NOG functional group in the samples. [file MBO3-14-e70208-s001.docx]

**Supplementary Information**

**Omics analysis of lignin degradation by the gut microbiomes of** **wood-eating** ***Hypomeces squamosus* Fabricius**

Chunlan Mao ^a,b,1*^, Qing Zhang ^c,1^, Jing Zhang ^c^, Xiangkai Li ^c^

^a^ State Key Laboratory of Ecological Safety and Sustainable Development in Arid Lands, Northwest Institute of Eco-Environment and Resources, Chinese Academy of Sciences, Lanzhou 730000, China

^b^ Lanzhou Eco-Agriculture Experimental Research Station, Lanzhou 730000, China

^c^ Ministry of Education Key Laboratory of Cell Activities and Stress Adaptations, School of Life Science, Lanzhou University, Lanzhou, China

^1^ The author has equal contributions and is regarded as the co-first author

* Corresponding Authors: Chunlan Mao; E-mail Addresses: maochunlan@nieer.ac.cn

**Supplementary Information:** 4 pages, 2 Tables, 2 Figures

**Table S1 Statistical table of metagenomics and meta-transcriptome assembly data**

| Sample | Parameters | Metagenomics | Meta-transcriptome |
| --- | --- | --- | --- |
| MHS_K | ORFS | 962747 | 6613 |
|  | Total length (bp) | 8.83E+08 | 3853863 |
|  | Max (bp) | 45702 | 10875 |
|  | Min (bp) | 200 | 60 |
| MHS_I | ORFS | 1409136 | 37666 |
|  | Total length (bp) | 9.55E+08 | 18549478 |
|  | Max (bp) | 75266 | 10981 |
|  | Min (bp) | 200 | 60 |

**Table S2 α-diversity analysis index of Metagenomic**

| Sample | simpson | shannon | chao1 | ACE |
| --- | --- | --- | --- | --- |
| MHS_I | 0.8712 | 4.0129 | 455.5 | 466.1713 |
| MHS_K | 0.8534 | 4.1565 | 438.0 | 414.2852 |

**
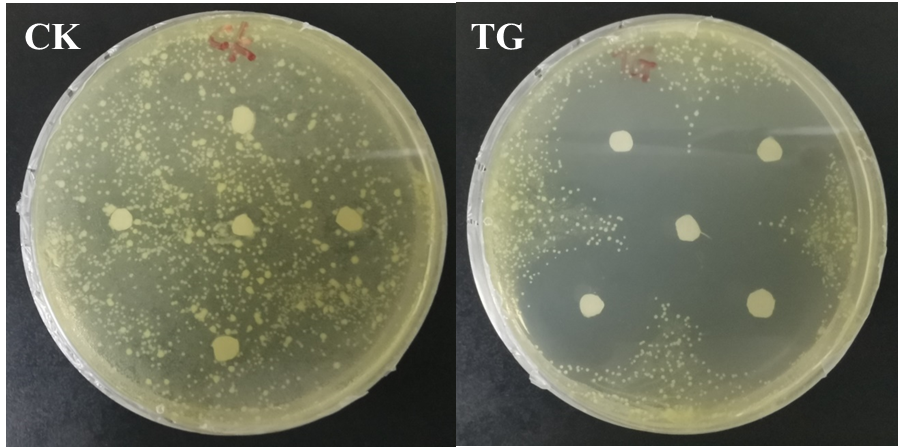
Figure S1 Inhibitory effect of gentamicin on the growth of bacteria extracted from the intestine of *Hypomeces squamosus* Fabricius.**

**
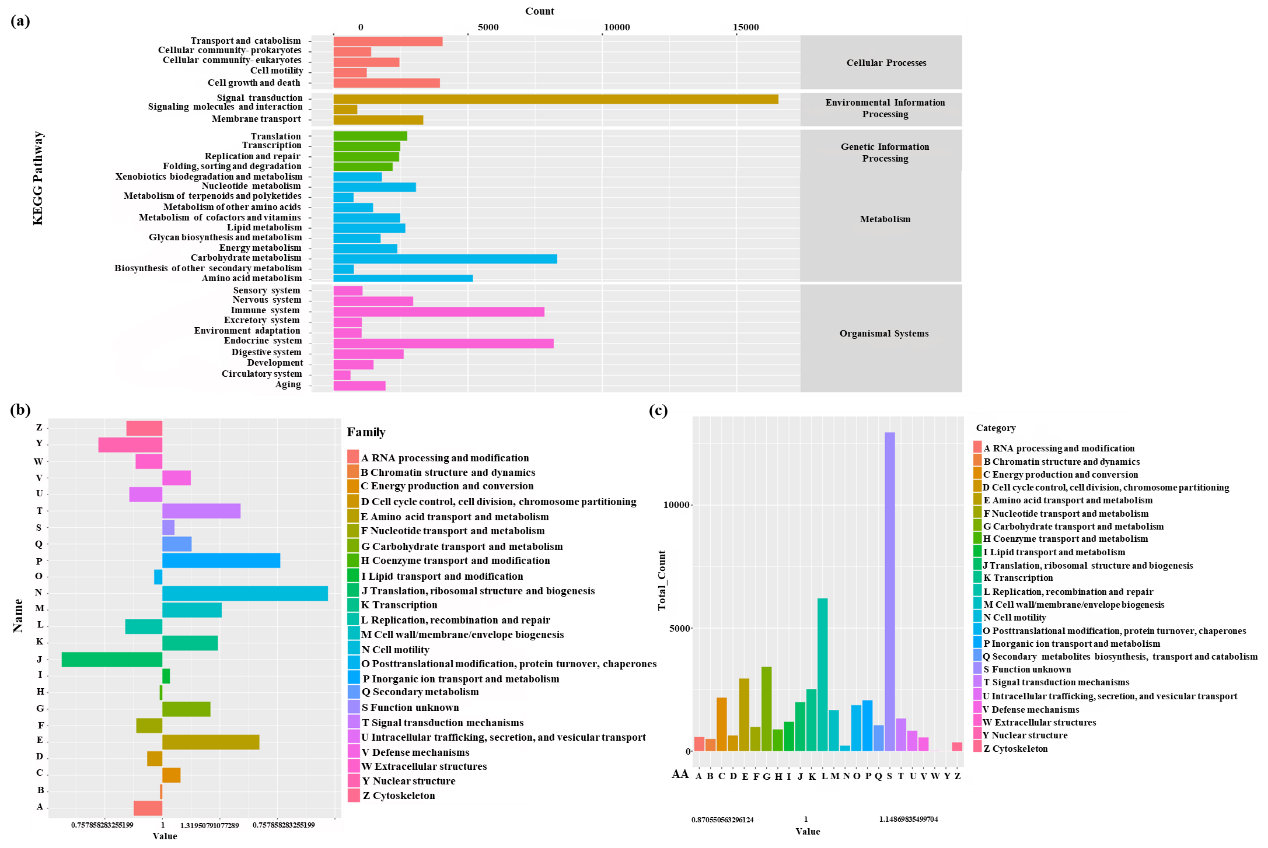
Figure S2 Functional annotations of metagenomic genes. (a) Statistical chart of annotation results of KEGG pathway at second level. (b) Annotated statistical graph of the EggNOG functional group. Comparison diagram of (c) NOG functional group in the samples.**
